# Outcomes and Experiences of Men and Women with Partner Notification for HIV Testing in Tanzania: Results from a Mixed Method Study

**DOI:** 10.1007/s10461-017-1936-x

**Published:** 2017-10-31

**Authors:** Marya Plotkin, Catherine Kahabuka, Alice Christensen, Denice Ochola, Myra Betron, Mustafa Njozi, Werner Maokola, Renatus Kisendy, Erick Mlanga, Kelly Curran, Mary Drake, Eusebi Kessy, Vincent Wong

**Affiliations:** 1 Jhpiego Tanzania, Dar es Salaam, Tanzania; 2 CSK Research Solutions Ltd., Dar es Salaam, Tanzania; 3 Jhpiego, Consultant, Baltimore, MD USA; 4 Jhpiego Baltimore, Baltimore, MD USA; 5 National AIDS Control Programme, Ministry of Health, Community Development, Gender, Elderly and Children, Dar es Salaam, Tanzania; 6 USAID Tanzania, Dar es Salaam, Tanzania; 7 Johns Hopkins Bloomberg School of Public Health, Baltimore, MD USA; 8 Ministry of Health, Community Development, Gender, Elderly and Children, Njombe, Tanzania; 9 USAID Washington, Washington, DC USA

**Keywords:** HIV testing, Partner notification, Index clients, Gender, Tanzania

## Abstract

A growing evidence base supports expansion of partner notification in HIV testing services (HTS) in sub-Saharan Africa. In 2015, a cross-sectional study was conducted in Njombe region, Tanzania, to evaluate partner notification within facility-based HTS. Men and women newly diagnosed with HIV were enrolled as index clients and asked to list current or past sexual partners for referral to HTS. Successful partner referral was 2.5 times more likely among married compared to unmarried index clients and 2.2 times more likely among male compared to female index clients. In qualitative analysis, male as well as female index clients mentioned difficulties notifying past or casual partners, and noted disease symptoms as a motivating factor for HIV testing. Female index clients mentioned gender-specific challenges to successful referral. Women may need additional support to overcome challenges in the partner notification process. In addition to reducing barriers to partner notification specific to women, a programmatic emphasis on social strengths of males in successfully referring partners should be considered.

## Introduction

According to the 2016 UNAIDS Global AIDS Update, there are an estimated 36.7 million people living with HIV, 54% of whom are not accessing life-saving treatment [1]. Effective approaches to HIV testing are needed to reach undiagnosed people and link them to HIV care and treatment. As part of the UNAIDS 90-90-90 goals, 90% of HIV-infected individuals will know their HIV status by the year 2020. However, few HIV testing approaches are highly effective in reaching undiagnosed HIV-infected people. A review drawn from Demographic and Health Surveys (DHS) from 2003 to 2011 noted that, in 16 of 25 countries studied, the majority of HIV-positive men and women have never been tested [2]. In Tanzania, 33% of women and 50% of men report never having been tested [3].

HIV testing services (HTS) in sub-Saharan African (SSA) countries have evolved as HIV prevention, care, and treatment interventions have changed. Between 2005 and 2007, most SSA countries began massive scale-up of HTS, and in 2007 the World Health Organization issued guidelines supporting provider-initiated testing and counseling (PITC), which recommended routine HIV testing in clinical settings [3]. By 2010, PITC policies were in place in a majority of SSA countries, with specific emphasis on testing within antenatal care services as prevention of mother-to-child transmission (PMTCT) services were rolled out [4]. However, HTS expansion in SSA has not been even. The massive scale-up of PMTCT services dramatically increased the number of women who learned their HIV status. A review of 29 SSA country DHS findings confirms that women are much more likely than men to have ever been tested [2]. Reaching men with HTS is thus a priority. A systematic review of strategies to increase the uptake of HIV testing among men noted that increased uptake of HTS by men is possible, and has been achieved, most notably through mobile and home-based HTS [5].

Partner notification, in which sexual partners of those recently diagnosed with HIV are notified of their exposure and referred to HIV testing, has shown promise in reaching previously undiagnosed individuals [6, 7] and has been shown to be well regarded by communities served by HIV testing services [8]. HIV partner notification is described in the 2015 World Health Organization (WHO) Consolidated Guidelines on HIV Testing Services, as a process in which persons newly diagnosed with HIV are enrolled as index clients, and then offered options for notifying and linking their sexual partners to HTS [9]. This was followed by WHO guidance released in 2016 which strongly recommended the offer of assisted HIV partner notification for all HIV-positive persons as part of HTS [10].

A growing base of evidence supports an expanded use of partner notification for HTS in SSA, where the majority of people living with undiagnosed HIV infection reside [11]. HIV partner notification has been shown to be effective in multiple countries. In Malawi, a randomized controlled trial compared passive, provider and contract referral to HIV testing of newly diagnosed HIV positive individuals, with an overall testing rate of 35% of partners (range 24–51%), of whom 64% were HIV positive [12]. An evaluation of a large scale partner services program in Cameroon found that 67% of partners tested, and 50% of them were HIV positive [13]. In Mozambique, there was a 99% acceptance rate of partner notification from community health workers, and only 2.5 index partners were needed to receive assisted partner notification in order to identify a previously undiagnosed partner with HIV [14]. In a cluster randomized trial in Kenya, 67% of partners had tested at 6 weeks after the index partners had enrolled in immediate partner notification services [15]. A recent meta-analysis concluded that partner notification improved partner testing and diagnosis of HIV positive partners, with few harmful consequences [7].

Multiple studies have shown that gender norms and roles have an impact on decisions people make around testing, partner notification of exposure, disclosure of HIV status and referral to HTS in sub-Saharan Africa [16–18]. While women‘s exposure to antenatal care services makes them more likely to be tested, they face a host of other barriers to HIV testing, status disclosure and/or partner notification that derive from gender roles and inequalities in relationships. These barriers may include lack of resources or ability to get to services; lack of education; and fear of violence, abandonment, or other abuse arising from partner notification [18–20].

For men, HIV testing and disclosure, particularly in SSA, may be inhibited by stigma and by social roles prescribing that men should be strong, dominant, and healthy. Multiple studies in Zimbabwe described men’s discomfort with testing, based on pressures related to gender roles and stereotypes [21, 22], and in Lesotho and Uganda, men described their fear of testing as a fear of showing weakness [23, 24]. A study in Zambia showed that gender determinants, including tolerant attitudes about intimate partner violence (IPV) and unequal power dynamics within relationships, had marked effects for men and for women, on the decision to seek HIV testing [16]. Studies have also found that men, compared to women, underestimate their level of risk of HIV infection [25]. However, once they have tested, men may be more likely to disclose their HIV status. A study of HIV-positive men and women in Burkina Faso showed that twice as many men as women reported having disclosed their HIV infection to their sexual partners, with some men indicating that they felt their role as the family’s breadwinner protected them against rejection when revealing their HIV-positive status following testing [26].

Differential outcomes of male versus female index clients in successfully referring partners to HTS in the context of partner notification in SSA have not been previously described in the literature. Further, although a recent publication addressed men and women’s experiences with disclosure of HIV status in South Africa [18], men and women’s experiences associated with partner notification has not been described. As partner notification for HTS is scaled up in SSA, gender and sex-related dimensions must be taken into account in implementation strategies, in order for partner notification and referral to HTS programs to achieve maximum impact.

This paper uses findings from a study in Tanzania to explore the research question: do male and female index clients have different outcomes in getting their sexual partners to come in for HIV testing following referral through the partner notification? The paper describes the success of men and women in referring their sexual partners to HTS in a partner notification study conducted in Tanzania, as well as describing barriers and experiences arising for index clients and their partners during the process of partner notification for HTS.

## Methods

### Study Design

A mixed-methods, cross-sectional study on partner notification for HIV testing was conducted in three hospitals in the Njombe region of Tanzania between June and September 2015. The overview results of the study, which looked at the effectiveness of partner notification in getting sexual partners to come to the facility for HIV testing, are described in a previous publication [27]. The current analysis examines referral outcomes among male versus female index clients. The main quantitative outcome of interest for this analysis was differences in success of referral for HTS among male versus female and between married and non-married index clients. The study also qualitatively explored men and women’s views on barriers and other experiences arising during the partner notification process, from both the index client and the sexual partner perspective.

### Study Setting

Njombe is Tanzania’s highest HIV prevalence region, with 14.8% of the adult population infected with HIV [3]. Study facilities included peri-urban Kibena Regional Hospital, urban Makambako Town Hospital, and the rural, faith-based Ilembula Designated District Hospital. Each facility had a dedicated, on-site voluntary counseling and testing (VCT) center, and offered PITC to inpatients and to outpatients. These three facilities were selected in consultation with regional authorities because of their high testing volume.

### Study Population and Eligibility Criteria

The study population comprised 390 index clients (men and women at the study facilities who had been diagnosed with HIV on the same day of testing, through either PITC or VCT) and 249 sexual partners of index clients (partners who had been listed by index clients and had come to the health facility for HIV testing). Eligibility criteria for study enrollment as an index client included the following: newly diagnosed with HIV; 18 years or older; not pregnant; had a sexual partner currently or in the past 24 months. Eligibility criteria for enrollment as a sexual partner was that the partner was 18 years of age or older. Pregnant women were excluded from enrolling in the study as index clients since a different form of partner notification exists in Tanzania within antenatal care services: pregnant women are encouraged to bring their partner into the health facility to be tested.

The 46 participants in the qualitative component of the study were selected on a convenience basis from the index clients and sexual partners enrolled in the study. The index clients were selected in equal number from male and female index clients who successfully referred a sexual partner and those who were not successful in referring a sexual partner, and the male and female sexual partners were selected in a roughly equal number according to whether they tested positive or negative for the virus. Index clients and partners were selected by study staff for participating in an in-depth interview (IDI) on a convenience basis. A rough quota was given to each facility, balancing out male and female participants, and those index clients or partners who were invited to participate and agreed and consented were interviewed.

### Sampling

The sample size for the study was calculated to answer the main study objectives, which was the successful referral of sexual partners of the index clients. The sample size calculation for the original research question was based on an assumption that index clients would list an average of one sexual partner, and that 51% of partners would come to the facility following notification, as seen in a study conducted in the hospital setting in Malawi [12]. Based on these assumptions, a sample size of 384 index clients was needed to detect a similar rate of attendance among sexual partners with 85% power (α = 0·05, two-sided test). The design effect (DEFF) was set at 1.0 because we expected minimal variation between facilities. The sample size formula was:

$$\begin{aligned} {\text{n }} = \, \frac{1.96^{2} {\text{p }}\left( {1 - {\text{p}}} \right) \, \left( {\text{DEFF}} \right)}{{\text{d}}^{2}} \, = \, \frac{1.96^{2} \times \, 0.51 \, \left( {1 - 0.51} \right) \, \left( {1.0} \right)}{\left( {0.05} \right)^{2}} \, = \, 384 \end{aligned}$$

The current analysis utilized this sampling framework. The differences between sex and marital status were thus not sampled for, but strongly emerged from the analysis.

### Study Procedures

#### Partner Notification

Potentially eligible men and women (diagnosed with HIV through PITC or VCT at the study facilities) were referred to onsite researchers at the time of their diagnosis. Study staff—who were trained HIV counselors—screened potential participants for study eligibility for enrollment as an index client (eligibility criteria detailed above). Written informed consent was obtained from interested and eligible people before enrolling them as index clients. Index clients wrote down names and contact information for sexual partners in a log, and these partners are referred to in this study as “listed sexual partners.” Enrolled index clients were interviewed using a questionnaire that collected demographic information and contained questions to flag individuals with a history of IPV. Index clients were asked to list current or past (within 24 months) sexual partners, provide locator information (most relevant being phone number), and decide how the partner was to be contacted for the referral to HTS. During partner listing, the index client was asked questions designed to identify listed sexual partners to whom disclosure or the referral to HTS might cause a risk of IPV. Any sexual partners the index client felt might react with violence were excluded from the notification process. Index clients could elect to contact the partner themselves (passive referral), have the study staff contact the partner (provider referral), or attempt to contact the partner themselves, with the understanding that the study staff would contact the partner should the index client fail (contract referral). A second written consent, was filled in before the index client listed partners, or before the study staff followed up with a partner. Index clients were requested to list as many partners as they could, with locator information, indicating the type (married, unmarried, casual partner, boyfriend or girlfriend), duration, and status (past or current) of the relationship for each partner. Index clients were given a choice as to whether or not they wanted a written referral card to take to their partner.

Sexual partners were referred back to the facility in which the index client tested (these referred partners are now no longer “listed sexual partners” and are referred to here as “sexual partners” or “partners”). Partners were given the name and room number of the counselor who had conducted the index client testing, and were offered HIV testing by the same counselor or another study counselor. This process both made it clear to the partner where to go as well as allowing the study staff to link the partner with the index client. Sexual partners who decided to seek testing at another facility, or who decided to come in for testing but did not identify themselves to the study staff, were not successfully referred partners in this study.

In this study, “successful referral” is defined as the partner coming to the health facility following referral (passive, contract or provider) whether or not the partner tested at the facility. A very small number of partners (n = 10) came to the facility but did not test due to being previously diagnosed HIV positive. These are counted as successfully referred since they came to the facility following referral.

#### Qualitative Interviews

IDI participants returned to the study site for the interview, and were reimbursed for their transport costs. Interviews were conducted in Kiswahili by a trained study staff member using a standardized interview guide. Interviews were audio recorded, transcribed into Kiswahili transcripts, and then translated into English.

### Data Management and Analysis

Quantitative data were collected either manually, using paper forms, or electronically, using tablets. Paper-based data were entered into ODK data files that had field checks for data quality. Data collected using tablets were uploaded immediately to a server in Dar es Salaam. Data were cleaned by running queries and reports using STATA version 14.0 and correcting discrepancies. Data were extracted and analyzed using SPSS version 23. Qualitative data were in the form of audio files, which were transcribed and translated, entered as English transcripts, which was then uploaded into MAXQDA (VERBI Software—Consult—Sozialforschung GmbH 2014).

Descriptive statistics were performed to describe the background characteristics of index clients and successfully referred sexual partners. Analysis entailed simple frequencies of the main study outcomes and cross-tabulations with Chi square tests to determine if there were significant differences in the success of referral for HTS among male and female index clients based on their background characteristics. Key analyses included differences in successful referral and listing of multiple partners by sex. Bivariate and multivariate logistic regressions were run to identify index client and partner characteristics associated with successful partner referral, including sex and marital/relationship status. Backward elimination was used to determine the final logistic model. Covariates were included in the model if 1) they had a p value < 0.25; and/or 2) were known to affect the outcome of interest from previously published studies. The main study outcomes in regression models were “listing more than one sexual partner” and “referring at least one sexual partner” for index clients (Table 2), and for sexual partners “being successful referred for HTS” and “having a positive HIV diagnosis” (Table 4). The covariates that were included in the model were age, sex, education and marital status.

For the qualitative data, a codebook was developed by a team of three researchers (DO, CK, MP) incorporating themes that corresponded to study interview guides. English transcripts were reviewed and uploaded into MAXQDA qualitative analysis software where the data were tagged using previously identified codes as well as codes that emerged from the data, in the Grounded Theory tradition [28]. One researcher applied cross-case analysis to organize the transcript data into themes and provided relevant narratives and quotations illustrating each theme [29].

**Fig. 1:**
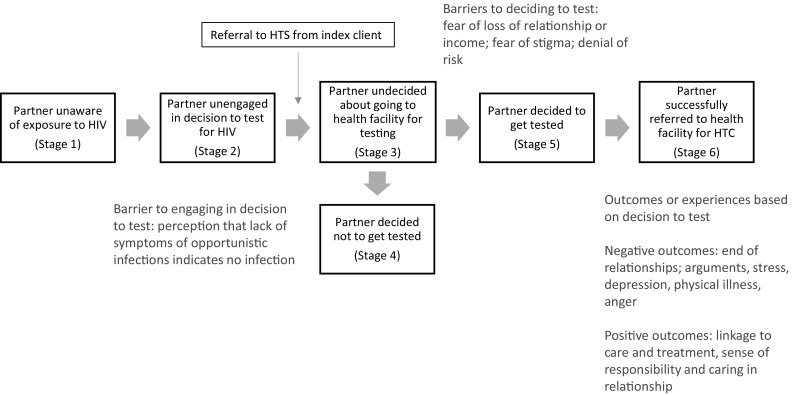
PAPM model with reference to partner notification process. Text in blue is based on findings from the current study (Color figure online). Adapted from Ref. [30]

In analysis of qualitative data, we adapted Weinstein et al.’s Precaution Adoption Process Model (PAPM) [30] to categorize the stages through which a partner might pass through in the decision of whether or not to come in for testing following a referral. The PAPM describes stages of precaution-taking, and seeks to elaborate the stages that people go through to commence a health-protective behavior [30]. This model, with some adaptations reflecting this study, can be seen in Fig. 1.

### Ethical Oversight

The study was conducted with ethical oversight from the Institutional Review Boards (IRB) of the Johns Hopkins Bloomberg School of Public Health (IRB 00006116) and the Tanzanian National Institute of Medical Research (NIMR) IRB (NIMR/HQ/R.8a/vol.1x/1914) with support from the Njombe Regional Medical Authorities.

## Results

### Results: Quantitative

#### Overview of Study Outcomes

**Fig. 2:**
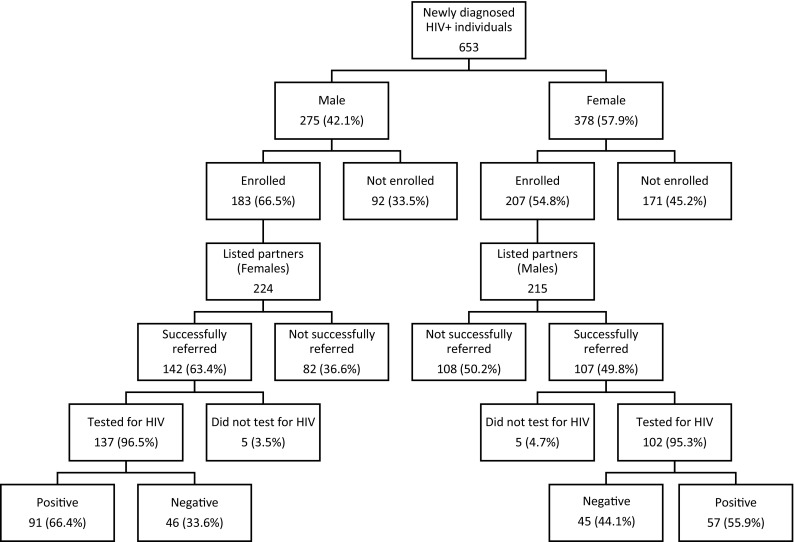
Overview, partner notification study enrollment, listing, referral and testing by sex, June–September 2015

Figure 2 depicts the number of HIV-positive individuals approached, enrolled index clients, and listed and successfully referred sexual partners by sex. Of the 653 newly diagnosed HIV-positive men and women, 390 (46.9% males and 53.1% females) were enrolled as index clients. The enrolled clients listed 439 sexual partners (224 female partners and 215 male partners). No index clients listed same-sex sexual partners. Of the listed partners, 249 (56.7%) were successfully referred (i.e., came to a study facility following the notification). Overall, 63.4% of the female partners and 49.8% of the male partners were successfully referred. Among partners who were successfully referred to the facility, testing was close to universal for both female and male partners (96.5% and 95.3%, respectively).

#### Enrollment and Eligibility

**Fig. 3:**
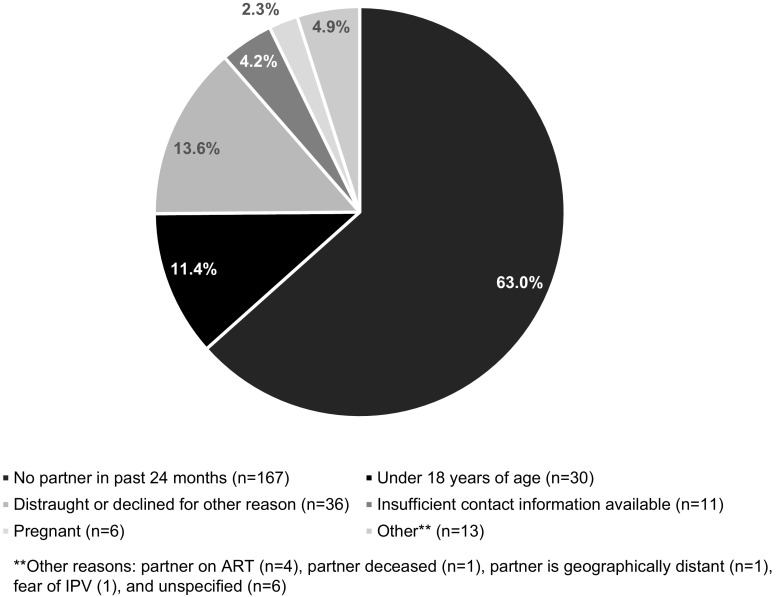
Reasons for non-enrollment in Tanzania partner notification study, June–September 2015

Enrollment and eligibility information is presented to augment understanding of the type of client seen in a typical service delivery setting who may or may not be interested or eligible for partner notification. Of the 653 HIV-positive individuals who were screened, 31 were ineligible since they were under 18 years of age, and 232 did not enroll in the study for other various reasons. The most frequently reported reason for non-enrollment was not having had a partner in the past 24 months (n = 167), followed by being distraught or decline to participate (n = 36), or other reasons (n = 13) (Fig. 3).

Table 1 presents demographic information on the 390 (enrolled) index clients and on the 232 newly diagnosed HIV-positive people above the age of 18 who did not enroll. Married people were much more likely to enroll in the study than unmarried people (76.2% vs. 24.8%).

**Table 1:** Demographic factors of HIV-positive, non-enrolled individuals and index clients

Demographic factors	HIV positive, non-enrolled in study (n = 232)				Index clients (n = 390)			
	Males N (%)	Females N (%)	Total N (%)	P-value	Males N (%)	Females N (%)	Total N (%)	p value
Age group				0.011				0.000
					NA	NA	NA	
18–24	4 (5.2)	31 (20.0)	35 (15.1)		13 (7.1)	49 (23.7)	62 (15.9)	
25–34	31 (40.3)	66 (42.6)	97 (41.8)		74 (40.4)	100 (48.3)	174 (44.6)	
35–44	25 (32.4)	33 (21.3)	58 (25.0)		61 (33.3)	41 (19.8)	102 (26.2)	
45 and above	17 (22.1)	25 (16.1)	42 (18.1)		35 (19.1)	17 (8.2)	52 (13.3)	
Education				0.757				0.541
No formal education	17 (22.1)	35 (22.6)	52 (22.4)		24 (13.1)	34 (16.4)	58 (14.9)	
Primary education	56 (72.7)	108 (69.7)	164 (70.7)		138 (75.4)	146 (70.5)	284 (72.8)	
Secondary and above	4 (5.2)	12 (7.7)	16 (6.9)		21 (11.5)	27 (13.0)	48 (12.3)	
Marital Status				0.375				0.000
Single	27 (35.1)	52 (33.5)	79 (34.1)		21 (11.5)	52 (25.1)	73 (18.7)	
Married	22 (28.6)	42 (27.2)	64 (27.6)		156 (85.2)	141 (68.1)	297 (76.2)	
Divorced	17 (22.1)	25 (16.1)	42 (18.1)		6 (3.3)	8 (3.9)	14 (3.6)	
Widowed	11 (14.2)	36 (23.2)	47 (20.2)		0 (0.0)	6 (2.9)	6 (1.5)	
Main economic activity								0.000
Housewife	NA	NA	NA		0 (0.0)	6 (2.9)	6 (1.5)	
Farmer					105 (57.4)	113 (54.6)	218 (55.9)	
Small scale business					39 (21.3)	72 (34.8)	111 (28.5)	
Formally employed					26 (14.2)	14 (6.8)	40 (10.3)	
Other					13 (7.1)	2 (1.0)	15 (3.8)	
Total	77 (100.0)	155 (100.0)	232 (100.0)		183 (100.0)	207 (100.0)	390 (100.0)	

*NA* information was not collected for the non-enrolled

#### Type of Referral and Relationship Status

Almost all index clients (91.6%) chose passive referral, meaning that they contacted their listed sexual partners themselves (data not shown). Table 2 shows the tendency of index clients to list multiple partners and success of partner referral, by sex and marital status of the index clients. Among index clients, 47 listed more than one sexual partner: in multivariate analysis, male index clients were 6.2 times more likely to list more than one sexual partner than female index clients. In multivariate analysis, male index clients were 2.2 times more likely than female index clients to be successful in referring at least one partner. In multivariate analysis, married index clients were 2.5 times more likely than unmarried index clients to be successful in referring at least one partner.

**Table 2:** Successful referral and multiple partner listing by sex and marital status of index clients

Study outcome	Index clients (N = 390)				
	Yes N (%)	No N (%)	Total N (%)	Bivariate Odds Ratio (CI)	Multivariate Odds Ratio (CI)^a^
Index client listed more than one sexual partner					
Female	10 (4.8)	197 (95.2)	207 (100.0)	Reference	Reference
Male	37 (20.2)	146 (79.8)	183 (100.0)	5.0 (2.4–10.4)*	6.2 (2.7–14.1)*
Index client listed more than one sexual partner					
Not married	11 (11.8)	82 (88.2)	93 (100.0)	Reference	Reference
Married	36 (12.1)	261 (87.9)	297 (100.0)	1.0 (0.5–2.1)	0.6 (0.3–1.3)
Index client referred at least one listed sexual partner					
Female	107 (51.7)	100 (48.3)	207 (100.0)	Reference	Reference
Male	130 (71.0)	53 (29.0)	183 (100.0)	2.3 (1.5–3.5)*	2.2 (1.4–3.5)*
Index client referred at least one listed sexual partner by marital status					
Not married	39 (41.9)	54 (58.1)	93 (100.0)	Reference	Reference
Married	198 (66.7)	99 (33.3)	297 (100.0)	2.7 (1.7–4.5)*	2.5 (1.5–4.2)*

*CI* confidence interval

* p < 0.001

^a^Adjusted for age, education, sex and marital status

#### Characteristics of Sexual Partners

Table 3 presents demographic information on the 249 successfully referred sexual partners. Successfully referred male partners tended to be older than female partners (27% vs. 9.2% were 45 years and above) (p < 0.00). While the majority of all partners (66.3%) had primary education, more male partners (73.8%) had completed primary education than female partners (60.6%) (p = 0.012). More of the male partners were formally employed than female partners (14% vs. 2.1%) (p < 0.000).

**Table 3:** Demographic characteristics of successfully referred sexual partners by sex

Demographic factors	Successfully referred sexual partners (n = 249)			p value
	Males N (%)	Females N (%)	Total N (%)	
Age group				0.000
18–24	11 (10.3)	30 (21.1)	41 (16.5)	
25–34	35 (32.7)	61 (43.0)	96 (38.6)	
35–44	32 (29.9)	38 (26.8)	70 (28.1)	
45 and above	29 (27.1)	13 (9.2)	42 (16.9)	
Education				0.012
No formal education	14 (13.1)	41 (28.9)	55 (22.1)	
Primary education	79 (73.8)	86 (60.6)	165 (66.3)	
Secondary and above	14 (13.1)	15 (10.6)	29 (11.6)	
Marital Status (missing information = 1)				0.124
Single	14 (13.2)	8 (5.6)	22 (8.9)	
Married/living together	89 (84.0)	131 (92.3)	220 (88.7)	
Divorced/separated	2 (1.9)	3 (2.1)	5 (2.0)	
Widowed	1 (0.9)	0 (0.0)	1 (0.4)	
Main economic activity				0.000
Housewife	0 (0.0)	3 (2.1)	3 (1.2)	
Farmer	61 (57.0)	102 (71.8)	163 (65.5)	
Small scale business/trader	21 (19.6)	30 (21.1)	51 (20.5)	
Formally employed	15 (14.0)	3 (2.1)	18 (7.2)	
Other	10 (9.3)	4 (2.8)	14 (5.6)	
Total	107 (100.0)	142 (100.0)	249 (100.0)	

Table 4 presents successful referral and HIV status of listed sexual partners by the sex, marital status, and relationship status of the listed sexual partners. In multivariate analysis, female sexual partners were 1.5 times more likely to be successfully referred than male sexual partners, and married partners 3.7 times more likely than unmarried. Partners who were listed as being a girlfriend/boyfriend or casual sex partner were less likely to be successfully referred: 30.4% of boyfriends/girlfriends and 33.9% of casual partners were successfully referred, compared to 72.5% wives and 54.7% of husbands.

**Table 4:** Successful referral and HIV status by sex, marital status, and relationship status of sexual partners

Study outcome	Yes N (%)	No N (%)	Total	Bivariate Odds Ratio (CI)	Multivariate Odds Ratio (CI)^a^
Partner successfully referred to HTS (n = 439 listed sexual partners)					
Sex					
Male sexual partner	108 (49.8)	109 (50.2)	217 (100.0)	Reference	Reference
Female sexual partner	141 (63.5)	81 (36.5)	222 (100.0)	1.7 (1.2–2.5)**	1.5 (1.0-2.2)*
Marital status (missing = 4)					
Unmarried	37 (32.2)	78 (67.8)	115 (100.0)	Reference	Reference
Married	208 (65.0)	112 (35.0)	320 (100.0)	3.9 (2.5–6.2)***	3.7 (2.3-5.8)***
Relationship status (missing = 5)					
Casual sexual partner	20 (33.9)	39 (66.1)	59 (100.0)	Reference	Reference
Husband	75 (54.7)	62 (45.3)	137 (100.0)	2.4 (1.3–4.5)**	1.8 (0.9–3.6)
Wife	132 (72.5)	50 (27.5)	182 (100.0)	5.1 (2.7–9.7)***	7.4 (3.3–16.3)***
Girlfriend/boyfriend	17 (30.4)	39 (69.6)	56 (100.0)	0.9 (0.4–1.7)	1.9 (0.9–4.4)
Partner diagnosed with HIV infection (n = 239 partners tested for HIV)					
Sex					
Male	57 (55.3)	46 (44.7)	103 (100.0)	Reference	Reference
Female	91 (66.9)	45 (33.1)	136 (100.0)	1.6 (1.0–2.8)	1.7 (0.9–3.3)
Marital status (missing = 5)					
Not married	17 (44.7)	21 (55.3)	38 (100.0)	Reference	Reference
Married	129 (65.8)	67 (34.2)	196 (100.0)	2.6 (1.3–5.4) *	1.5 (0.6–3.6)
Relationship status (missing = 5)					
Casual sexual partner	13 (65.0)	7 (35.0)	20 (100.0)	Reference	Reference
Husband	42 (60.0)	28 (40.0)	70 (100.0)	0.8 (0.3–2.3)	0.3 (0.1–1.6)
Wife	88 (69.3)	39 (30.7)	127 (100.0)	1.2 (0.5–3.3)	0.5 (0.1–3.1)
Girlfriend/boyfriend	3 (17.6)	14 (82.4)	17 (100.0)	0.1 (0.0–0.5)**	0.1 (0.0–0.5)**

*CI* confidence interval

* p < 0.05

** p < 0.01

*** p < 0.001

^a^Adjusted for age, sex, education and marital status

### Results: Qualitative

A total of 46 participants (26 index clients and 20 sexual partners) were interviewed as part of the IDI’s. Half were male and half female. Table 5 presents demographic characteristics of the IDI participants.

**Table 5:** Demographic characteristics of in-depth interview (IDI) participants

Demographic characteristics	Index clients		Sexual partners		ALL IDI Participants (n = 46)
	Successfully referred at least one partner (n = 12)	Failed to refer a sexual partner (n = 14)	Testing positive (n = 9)	Testing negative (n = 11)	
Sex					
Male	5	7	4	7	23
Female	7	7	5	4	23
Age					
18–24	1	0	0	3	4
25–34	3	7	5	2	17
35–44	6	2	2	3	13
45 and above	2	5	2	3	12
Highest Education					
No formal education	1	2	6	8	17
Primary education	10	10	1	1	22
Secondary education and above	1	2	0	1	4
Missing information	0	0	2	1	3
Marital Status					
Single	3	4	0	3	10
Married	9	9	9	6	33
Divorced/separated	0	0	0	2	2
Widowed	0	1	0	0	1
Total	12	14	9	11	46

#### Language Used in Notifying Partner

Many of the index clients interviewed chose to use indirect language (i.e., avoided direct reference to HIV testing or avoided disclosing that they had been tested) when notifying and referring their partner. Multiple male index clients interviewed told their partners that the health care provider would not reveal their test results unless the partner came for testing as well. Some female index clients pretended they had not yet tested, or claimed to have a health problem for which their partner’s assistance was needed.

Index Client KRH-068/no trace/M/40/Married: To be honest… I did not want to tell her. I tried to convince her, by telling her that “I will not get my results until I go with you,” She wanted to know why do others go there and get their results? I told her that is for those who are not married. I insisted that I have gone there but I could not get the results without going with her: that is where we ended our conversation.

Index client MTH203/trace/F/39/Married: I called him, I told him that you are needed here; he asked “what for!?” I told him to come because they have told me that I have anemia, “You have to come, for you to get the information by yourself; they need to tell you many things,” so he agreed to come…. [To what extent was the way you used effective?] Very effective… .

Partner IDDH-020-1/-ve/M/19/Single: *(Liked the indirect method of notification his partner used)* I think it was a right method, because the way she told me she has a problem without telling me what it is, made me anxious to know what is it.

Some index clients, however, told their partners directly that they had tested positive during the referral to HTS. Generally, partners who were informed directly expressed appreciation for the candid disclosure:

Partner MTH-119-01/+ ve/F/30/Married: “I was encouraged when he told me: “Myself, I am affected so I ask you to go and get tested”…. I was encouraged because he told me openly.”

Index client MTH-132/trace/F/29/Living together: I told him that I have tested already and the results are known…. He told me that what I did was good. He told me yes, it’s true we both need to go for test; I need also to know my status.

Index client IDDH-009/M/age/Unmarried: (*Female partner suggested index test due to continuing illness. He was afraid of her reaction to the results. The second time he talked to her, he told her that she needed to come to the facility to talk to the health care provider. When she guessed that he was positive, he hung up the phone.*) She phoned me back and said, “Don’t worry, if you’re positive just tell me. You should not be afraid.”

#### Barriers to Referral of Partners for Testing or Partner Notification

##### Time/Distance as a Barrier

 Many index clients made contact with their partners over the phone. Index clients—those who were successful in referring partners as well as those who were not—mentioned barriers involving time and geographical distance.

Index client MTH122/trace/M/42/Married: [Why were you not able to remember most of your partners?] Because these people, I was with them in a far off location from here, I think I got this problem [HIV infection] not here, but there in Madibira. That’s why I was not able to remember all of them.

Some female index clients noted that male partners whose jobs required frequent travel were particularly difficult to locate and/or refer.

##### Bad End or Long Duration of Separation as a Barrier

 Male as well as female index clients were reluctant to get in touch with former partners who were suspected, or were known, to have married, or with whom the relationship had ended poorly.

Index client KRH-042/trace/F/43/Married: [For the partner you could not convince to come for testing, what do you think was a major obstacle?] I could not, because those [relationships] are from a long time ago, since the year before last year. Would you call someone who you had broken up with? The person has a wife or wives and you broke up with a fight. You do not call each other! Why would you call such a person?!

Index client IDDH-009/trace/M/36/Married: As I told you earlier we separated, not in a peaceful way, it wasn’t in a manner that we could later look for one another. She moved on and got married, and had a child with someone else.

##### Absence of Symptoms as a Barrier

 Many index clients reported that either they or their partners did not feel they should test for HIV because of a lack of symptoms of opportunistic infections. In some cases, the presence of symptoms prompted testing-seeking behavior in the partner; in some cases it motivated the index client to seek testing. Alternatively, the absence of symptoms served as justification for the decision not to test, for male partners in particular.

Index client MTH-107 no trace/F/26/Married (bar worker): They [my partners] have refused; I am still in the process of mobilizing them to come [for testing]…. Both replied that they are not ready…. The first one said he is confident that he is healthy, and the other one… he is not ready to test until he gets sick.

Symptoms were mentioned in numerous cases as the reason that the index client sought HIV testing.

Index client MTH-148/no trace/M/54/Married: So those diseases were troubling us, she was complaining sometimes about back pain, sometimes stomach ache. Sometimes we feel hungry, she cooks but we can’t eat…. She said, “My husband, when you go there you should check this [HIV].”

Index client IDDH-007/trace/F/37/Married: I got that courage because I was sick… and it seems this was the root cause. That is why I had the courage to talk to him. I said I can’t keep on being afraid, as am already infected and I am the one suffering.

#### Type of Relationship and Success in Referral

When index clients discussed casual or multiple concurrent partners, they characterized this as difficult for partner notification or expressed reluctance to engage in partner notification:

Index client MTH-090/no trace/F/28/Single: [Were you able to remember all of the partners you had within 24 months?] Really I can’t recall them…. Even if I recall them it won’t be easy for me to give them this information… because others have their own families already. We just met as “friends for a day.” You can meet someone when traveling or at work. If you met someone just for a day, do you think you can tell him this matter?

Index client MTH-060/no trace/M/30/Married: So I told them [my partners] that I have checked and I have been found with problems, please you must also go for a checkup. One of them said you have told me a very important thing. But another one felt I did something wrong. [What did she say?] She said what is yours is yours and what is mine is mine…. [Are you living with one of those three or they are just sex partners?] These are my sex partners, apart from my wife. I told my wife, but she did not say anything.

Multiple women brought up the issue of having a child with their partner. If a woman did not have a child with her male partner, this was often presented as a less serious relationship or as a problematic relationship with poorer success at partner notification. The theme in this quote was echoed in multiple other female index clients’ quotes:

Index client IDDH-011/no trace/F/29/Single: [What stopped him from coming into test?] Maybe he wasn’t interested in me…. He once told me the way I am sick, “I’m tired of spending my money, because you mean nothing,” because I didn’t bear him a child…. [Therefore, the main reason is a child.] Yes, he said he can’t take care of me every time because I don’t have a child.

Two female index clients indicated that they were engaged in commercial sex as a livelihood, one married and one unmarried. These women suggested that commercial sex work made partner notification very difficult.

Index client MTH-107/no trace/F/26/Married: [Do you think you can remember all of your partners within the past 24 months?] No, I can’t remember them. [Why?] Because of my working environment… in a bar. There are those you see them once and never see them again. Up until now I only have two whom I have here with me.

#### Negative Outcomes to Partner Notification

None of the index clients or partners interviewed reported being physically hurt as a result of their participation in the notification or referral process, and none of the participants in the qualitative assessment reported cases of IPV. However, four of the interviewed female index clients (one married and three unmarried) mentioned, at the time of the interview, that the HIV diagnosis resulted in the end of the relationship.

KRH-053/trace/F/23/Single (engaged): We entered in the counseling room and had a health checkup. Then he tested and my partner was found clean, while on my side there was a problem. After that we decided to have a meeting…. I told him finally, I’m ready to break up and you are free to marry another woman if you decide…. And that was the end of the relationship.

MTH-090/no trace/F/28/Single: I don’t know what he is thinking but what I know is one day he will go to test voluntarily. Right now is like I am forcing him. He is rude. Since I told him about going to test we are not in good terms, we are not close anymore, we don’t see each other, it’s like he is not around. He doesn’t call me… just because I told him about testing.

Only one male index client (MTH122/trace/M/42/Married) noted strong negative feedback, and this was from his in-laws rather than his sexual partner.

Female as well as male sexual partners interviewed experienced stress-related physical and/or psychological distress after being notified of their potential HIV exposure.

Partner MTH-076-1/-ve/M/42/Married: It was difficult… for sure you cannot feel good… you feel as if you don’t deserve to live.

Partner KRH-001-1/+ve/F/35/Married: I got stomach problems since I was shocked to the extent that I experienced diarrhea…. But I was encouraged later. I continued to recover…. I reached a point where I had to encourage myself that I should persevere. I will be treated… .

#### Precaution Adoption Process Model (PAPM) for Partner Notification

When applying the (PAPM) (Fig. 1) [30] to the process of partner notification evaluated in this study, we found that the equivalent to Stage 1 was when the partner notification process made sexual partners aware of their exposure to HIV. Following notification, many partners reported a period of shock or distress, during which they were unable or unwilling to contemplate getting tested (Stage 2). For partners who did not test, it is unclear whether they stayed at Stage 2, unengaged in the decision of whether to test for HIV, or if they went through the decision-making process and ended up at Stage 4 (decided not to test). We were not able to interview any partners who did not come in for testing. Our analysis centered around those partners who reached Stage 6, successful referral. The quantitative and qualitative data both show that the decision to test (Stage 5) and successful referral (Stage 6) were heavily influenced by the relationship between index clients and their sexual partners (specifically whether or not they were married), with married partners as the group most likely to reach Stage 6. The PAPM highlighted the decision-making process around testing for HIV faced by partners, influenced by barriers and facilitators, some sex and gender-related, some of which were described to us by participants in the study.

## Discussion

In this model of partner notification (integrated into routine HIV testing, high prevalence HIV region) index clients’ ability to successfully refer partners to testing significantly varied based on marital status and sex of the index client. Married index clients were 2.5 times more likely than unmarried index clients to be successful in referring at least one sexual partner, and male index clients were 2.2 times more likely than female index clients to get at least one sexual partner to come in for HIV testing. Although not a predictor of success, it is notable that male index clients were 6.2 times more likely to list more than one sexual partner.

Many of the stated barriers to successfully referring partners for testing were not specific to sex or gender-related. These included geography (meaning partners were remote or traveled for work) and the absence of symptoms in partners. Past partners, multiple/casual partners, and partners in relationships that ended badly were all mentioned by both male and female index clients as people whom the index client would be reluctant, or unable, to contact. Both male and female sexual partners discussed experiencing anger, depression, physical ailments such as nausea and lack of appetite, stress and exhaustion around the HIV testing.

Some barriers to partner notification appeared to impact women more heavily than men. Experiences mentioned only by women included being abandoned or the ending of the relationship because HIV status was disclosed; being undervalued for not having a child; and having difficulty in notifying partners when her livelihood was sex work. These findings, which point to gender barriers which women disproportionately face, echo findings from other studies. In a study from South Africa, unequal and gendered dynamics in relationships caused different likelihood of HIV status disclosure when comparing men to women [18].

Globally, men are less likely than women to engage in each stage of the testing and treatment cascade [31]. However, our findings also showed that men had more success in referring partners. Sex and gender power differentials have long been associated with HIV-related risk factors, decision-making, including around testing, and consequences associated with status disclosure. In Uganda, a study showed that men may be more susceptible to perceptions about community norms for testing for HIV than women, since men’s perceptions that testing was “not normal” was associated with never testing [32]. Multiple reviews have shown that, for women, fear or experience of physical or emotional violence or abandonment upon disclosure of HIV status are very real concerns, and these concerns may impact women’s decisions regarding testing [19, 20]. Qualitative findings from this study indicate that for some participants these were also concerns. This study did not measure or analyze male or female gender norms, attitudes or power dynamics in relationships as they relate to willingness to test or ability to refer partners. However, other studies have found evidence for these impacting HIV care-seeking, treatment and disclosure [21, 33].

Based on study findings, a targeted effort to introduce men to HIV testing and increase their engagement in partner referral may have tremendous potential for finding previously undiagnosed people with HIV infection. Such efforts will require working against social and health system patterns in which women attend health services much more than men, and also against male social norms that undermine testing [33]. However, evidence suggests that interventions which challenge masculine norms and promote gender equality (gender-transformative interventions) can foster greater willingness to be tested for HIV and initiate care and treatment for those testing positive [33]. Supportive social networks can also increase a man’s likelihood to test: a recent study in Tanzania found that men who thought at least one close friend in their social network had ever tested were 2.7 times more likely to have ever been tested for HIV themselves [34]. A study in Uganda suggested that billboards or radio messages that disseminate information on true HIV testing uptake in communities could affect uptake of HTS by men [32]. Further research is needed to understand how best to attract men to testing services or reach them in communities. Helpful suggestions, including multi-session interventions and interventions focused on trustful communication between couples, were made in a recent study on HIV status disclosure in South Africa [18]. Different strategies may be needed for notifying partners who are considered hard to reach, such as former partners or partners of index clients who have multiple casual partners. According to a study in Uganda which included sex workers, provider assisted approaches may be more acceptable to people who have multiple casual partners, such as female sex workers or fishermen [8].

In our study, no index clients or partners reported physical abuse. Partners as well as index clients described negative consequences such as sadness, anger, disturbance in sleep, anxiety, headaches, diarrhea, not being able to eat, and in the case of four female index clients, the dissolution of marriages or relationships. This is consistent with a recent systematic review of partner notification approaches, which showed very few cases of harm, leading to WHO’s 2016 recommendation to include assisted partner notification in HIV testing and care services [32].

This study did have some limitations. Our sample for the qualitative component of this study was a convenience sample and thus may not represent the experiences of all the index clients and partners involved in this study. Although the qualitative findings highlighted both differences and similarities in experience between male and female participants in the partner notification process, it was not designed to elicit in-depth findings around gender roles and norms in relation to partner notification. In regards to the high number of HIV-positive individuals ineligible because they did not have a partner in the last 24 months, we feel that it is quite likely that some HIV-positive individuals may have been using this response as a way to politely opt out of the partner notification process. Despite this, the acceptance rate is consistent with findings from the region: in our sample, 25.6% of the HIV-positive people approached indicated they had not had a sexual partner in the past 24 months, while in the 2011–2012 Tanzania HIV/AIDS and Malaria Indicator Survey, 75.2% of male and 72.1% of female respondents in Njombe region indicated that they had a sexual partner in the last 12 months [3]. An amendment to the eligibility criteria for partners occurred after the first month of fieldwork. At that time, we extended the criteria for eligibility from a sexual partner within the last 12 months to a sexual partner within the last 24 months, because many participants indicated that they had partners within the last 24 but not 12 months. This may have affected roughly 20% of our index clients’ listing and notification choices, and may have had the effect of slightly limiting number of index clients participating and number of partners listed in the initial month of the study. Additionally, this study was not designed to capture testing which occurred outside of the three study facilities. If partners went to another facility to test following referral or if they went to the same facility but did not identify themselves to study staff, we would not know about that partner’s decision to test. This could mean that our estimates of the successful referrals in the study are lower than the actual. Finally, our study was not designed to assess the safety of the partner notification approach in regard to IPV. We recognize that even though no cases of IPV were reported, unreported cases may have occurred.

Our findings on the sex-related differences that create barriers for partner notification and HIV sero-status disclosure underscore the importance of principles described in the World Health Organization tool for integrating gender into HIV/AIDS programs in the health sector [35]. Programs scaling up partner notification should consult this tool closely. For example, by discussing the benefits and potential disadvantages of disclosure, programs can help women disclose their HIV status safely. Programs can also help those at risk of violence with safety planning and mediated disclosure [35].

## Conclusion

Partner notification integrated into routine facility HIV testing, in this high HIV-prevalence setting in Tanzania, was an effective way to reach previously undiagnosed HIV-infected individuals. Marital status was a huge determinant of success in referral, with married index clients 2.5 times more likely to be successful in referring their sexual partners to HTS. Success at referral to HTS varied by sex, with men being 2.2 times more likely to succeed in referring at least one sexual partner. Major barriers were both non-sex and gender-specific (being reluctant to contact a previous partner who is in another relationship, having a short-term relationship and being uncomfortable contacting the partner) and sex and gender–specific (feeling undervalued in a relationship because of the absence of childbearing, fear of dissolution of the relationship, which were greater concerns for females). It is clear that partner notification is deeply entrenched in roles and norms related to sex of the index client as well as norms related to marital status. Programs which scale up partner notification for HTS should take these factors into consideration in development of messages for clients in partner notification services. Formative studies of gender dynamics and gender-related barriers and facilitators of partner notification for men compared to women, including studies of both attitudes to and experiences with IPV, are recommended either before partner notification programs are rolled out or associated with rollout. Monitoring systems should be designed to capture successful referral to HTS by sex, age, marital status, so as to give useful feedback on these different characteristics to program planners. Additionally, partner notification service planners and implementers should be aware that reaching non-marital partners, and supporting female index clients to refer their sexual partners to HTS, may require additional investment of time and resources.
